# Special Issue: Human Movement Monitoring Using Wearable Sensor Technology

**DOI:** 10.3390/s25010208

**Published:** 2025-01-02

**Authors:** Yi-Chung Lin

**Affiliations:** 1 Sports Performance, Recovery, Injury and New Technologies (SPRINT) Research Centre, Australian Catholic University, Fitzroy, VIC 3065, Australia; yilin@acu.edu.au; 2 School of Behavioural and Health Sciences, Australian Catholic University, Fitzroy, VIC 3065, Australia

The accurate and reliable measurement of human movement stands as a cornerstone in the evaluation of athletic performance, the management of fall risk, the prevention of injury, and the guidance of clinical rehabilitation. This field’s scientific foundations were laid in the late 19th century when British photographer Eadweard Muybridge (1830–1904), a pioneer of cinematography, revolutionized motion analysis through his groundbreaking collection of 20,000 photographs capturing both animal and human movement [1].

The 20th century witnessed rapid advancement in human movement science, marked by the emergence of optical motion capture systems. These systems, initially developed for biomechanics research and clinical gait analysis in academic and medical settings [2,3,4], underwent significant commercialization in the latter part of the century, enabling unprecedented precision in motion analysis [5]. However, traditional optical motion capture systems remain tethered to laboratory environments, requiring controlled lighting conditions and time-consuming calibration processes that limit large-scale data collection.

The advent of wearable sensor technology has transformed this landscape, offering a versatile, non-visual approach to motion tracking that transcends indoor–outdoor boundaries [6]. These sophisticated devices, whether integrated into clothing, worn as accessories, or attached directly to the body, capture an extensive range of physiological and kinematic data. By measuring parameters such as acceleration, angular velocity, heart rate, and muscle activity, wearable sensors provide unprecedented insight into human movement patterns and dynamics. This technological breakthrough has catalyzed innovations across multiple domains, from comprehensive gait analysis and athletic performance optimization to revolutionary approaches in rehabilitation medicine and movement disorder diagnostics [7].

As we conclude this Special Issue, we celebrate the diverse contributions that advanced our understanding and technological capabilities in human movement monitoring. Fang et al.’s systematic review of methods for converting inertial measurement unit (IMU) data into joint angles for upper-limb movement quantification provides a comprehensive overview of the advancements and challenges in accurately measuring 3D motion. Their findings highlight the importance of computational methods and the potential for future innovations to overcome current limitations, paving the way for more precise and reliable joint angle estimation using wearable sensors.

The other 15 contributions in this Special Issue are organized into the following six main research areas: (1) Sports Performance and Training, (2) Running Biomechanics, (3) Human Activity Recognition, (4) Manual Wheelchair Propulsion, (5) Technological and Methodological Advances, and (6) Health Monitoring. These contributions offer valuable insights and present advances that will help shape the development and optimization of next-generation mobile systems.

Sports Performance and Training

1. Andriolo et al.’s study explores the relationship between cycling power and the short-term scaling exponent alpha1 of detrended fluctuation analysis (DFA-a1) of heart rate variability using everyday workout data from the AI Endurance app. The findings reveal a consistent link between cycling power and DFA-a1, suggesting the potential for real-time intensity zone tracking.
2. Radu et al.’s investigation into the effectiveness of MyVert technology in improving jump shot performance in young basketball players highlights the significance of sensor-driven feedback in youth sports training. Their findings suggest that a combined program of plyometric and coordination exercises, guided by MyVert’s data, can lead to significant physical and technical improvements.
3. Michailidis examines the relationship between aerobic capacity and running performance in youth football players during small-sized games on varying pitch sizes. By examining correlations between aerobic capacity and external/internal load measured by GPS devices, their findings suggest that aerobic capacity has a greater impact on running performance on larger pitches.
4. Chen et al.’s study on the relationship between overhead squat performance and golf swing kinematics offers a unique perspective on the application of wearable sensors in golf biomechanics. Golfers with better overhead squat ability showed greater sagittal plane extension, reduced lumbar angular velocity, and lower L4-S1 joint shear force, indicating that the overhead squat test can be a valuable tool for reducing the risk of golf-related lower back injuries.

Running Biomechanics

1. Lin et al.’s research on IMUs demonstrates their effectiveness in capturing lower-limb kinematics and pelvic orientation during high-speed running. Their comprehensive validation study provides valuable insights into the capabilities and limitations of the present IMU system in terms of measuring running kinematics.
2. Pirscoveanu and Oliveira assess the sensitivity of smartwatch-derived variables to changes in vertical impact loading during running, highlighting the limitations and future potential of consumer-grade fitness technology in biomechanical analysis.

Human Activity Recognition

1. Khan et al. propose a federated learning framework that combines spiking neural networks and long short-term memory (LSTM) models to enable energy-efficient and privacy-preserving human activity recognition. Evaluations of datasets collected from smartphones showed the proposed hybrid spiking–LSTM model outperformed LSTM, CNN, and S-CNN models in federated settings.
2. Yu et al.’s research on extracting dynamic balance variables from gait via a single IMU using recurrent neural networks demonstrates the potential of wearable sensors in fall risk management for older adults. Their novel approach provides a valuable tool for monitoring and assessing balance stability in this vulnerable population.

Manual Wheelchair Propulsion

1. Aissaoui et al.’s research on estimating handrim forces and moments using a single IMU and non-linear modeling offers a practical and efficient approach to monitoring the loads experienced by wheelchair users. Their method offers a more practical and less intrusive way to monitor the forces experienced by wheelchair users in real-world settings, potentially aiding in the prevention of upper-limb injuries.
2. Sipes et al.’s development of a machine learning model to predict external loads during handcycling is a significant step towards creating tailored exercise guidelines that minimize injury risk while promoting cardiovascular health. Their research demonstrates the potential of using inertial measurement units to optimize handcycling training programs.

Technological and Methodological Advances

1. Kim et al.’s innovative method of using deep learning to estimate 3D ground reaction force from shoes equipped with uniaxial load cells demonstrates the potential for enhancing the capabilities of wearable sensors. Their research offers promising applications for outdoor gait analysis and the non-invasive assessment of movement patterns.
2. Xia et al.’s optimization of a torque control model for quasi-direct-drive knee exoskeleton robots using regression forecasting showcases the intersection of wearable technology and robotics. Their research addresses key challenges in human–machine interaction and offers ways to improve the functionality and efficacy of assistive devices.

Health Monitoring

1. Emmerzaal et al.’s research on upper-limb movement quality in breast cancer patients before and after surgery underscores the importance of wearable sensors in monitoring post-operative recovery. Their findings highlight the need for more refined metrics to track rehabilitation progress and assess functional outcomes.
2. Dos Santos et al.’s evaluation of smartphone-based Timed Up and Go tests demonstrates the practicality and reliability of using mobile technologies for fall risk assessment. Their research highlights the growing role of wearable sensors in enabling more precise and accessible health monitoring.
3. Bai et al.’s novel approach using a transformer algorithm and biosignal fusion offers a more accurate and robust method for estimating joint acceleration in the shoulder and elbow. Their research demonstrates the power of advanced signal processing techniques in biomechanical assessments of upper-limb movements.
